# Quantifying the Trajectory Tracking Accuracy in UGVs: The Role of Traffic Scheduling in Wi-Fi-Enabled Time-Sensitive Networking

**DOI:** 10.3390/s26030881

**Published:** 2026-01-29

**Authors:** Elena Ferrari, Alberto Morato, Federico Tramarin, Claudio Zunino, Matteo Bertocco

**Affiliations:** 1 Department of Information Engineering, University of Padova, 35131 Padova, Italy; elena.ferrari.7@phd.unipd.it (E.F.); federico.tramarin@unipd.it (F.T.); matteo.bertocco@unipd.it (M.B.); 2 National Research Council of Italy—IEIIT, 35131 Padova, Italy; 3 Department of Management and Engineering, University of Padova, 36100 Vicenza, Italy; 4 National Research Council of Italy—IEIIT, 10129 Torino, Italy; claudio.zunino@cnr.it

**Keywords:** time-sensitive networking (TSN), wireless TSN, IEEE 802.1Qbv, trajectory tracking, unmanned ground vehicles (UGVs), traffic scheduling, real-time communication, mobile robotics, networked control systems, Wi-Fi TSN

## Abstract

Accurate trajectory tracking is a key requirement in unmanned ground vehicles (UGVs) operating in autonomous driving, mobile robotics, and industrial automation. In wireless Time-Sensitive Networking (WTSN) scenarios, trajectory accuracy strongly depends on deterministic packet delivery, precise traffic scheduling, and time synchronization among distributed devices. This paper quantifies the impact of IEEE 802.1Qbv time-aware traffic scheduling on trajectory tracking accuracy in UGVs operating over Wi-Fi-enabled TSN networks. The analysis focuses on how misconfigured real-time (RT) and best-effort (BE) transmission windows, as well as clock misalignment between devices, affect packet reception and control performance. A mathematical framework is introduced to predict the number of correctly received RT packets based on cycle time, packet periodicity, scheduling window lengths, and synchronization offsets, enabling the a priori dimensioning of RT and BE windows. The proposed model is validated through extensive simulations conducted in an ROS–Gazebo environment, utilising Linux-based traffic shaping and scheduling tools. Results show that improper traffic scheduling and synchronization offsets can significantly degrade trajectory tracking accuracy, while correctly dimensioned scheduling windows ensure reliable packet delivery and stable control, even under imperfect synchronization. The proposed approach provides practical design guidelines for configuring wireless TSN networks supporting real-time trajectory tracking in mobile robotic systems.

## 1. Introduction

In the field of tele-guidance, autonomous driving, mobile robotics, and industrial automation, precise and accurate trajectory tracking have become a fundamental requirement. In these scenarios, particularly those requiring autonomous and unsupervised operations, the ability to follow a predefined trajectory with high accuracy is essential to ensure both the safety and efficiency of the system.

The control of the trajectory of an Unmanned Ground Vehicle (UGV) can be divided into two fundamental aspects. The first relates to the detection, localization and, when necessary, the positioning of the robot within its environment. The second refers to the precision and accuracy with which the robot follows a predefined trajectory [1].

Although the first aspect focuses on the sensors used and their underlying technologies, such as Ultra-Wideband (UWB), GPS, and others, the actual accuracy of the trajectory tracking depends on a variety of factors. These include the control algorithm, the communication network, signal latency, and environmental conditions. Specifically, in distributed applications, the communication network plays a critical role in ensuring timely exchange of information between the robot and the control station. This is particularly important in robotic scenarios where the use of the Robotic Operating System (ROS) or additional Best-Effort (BE) traffic introduces additional data that must be effectively managed.

Moreover, traffic scheduling becomes vital when multiple kind of traffic are involved in the system, i.e., Real-Time (RT) and BE. In such applications, traffic must be scheduled at precise time intervals to not affect the performance of the system, thus guaranteeing that the RT traffic is not affected or delayed by the BE one. In fact, if RT and BE traffic are not properly handled, this can lead to unexpected behaviors or reduced accuracy in control actions, such as trajectory tracking, since not all the frames could be correctly received. To address these unsolved challenges of such application scenarios and create more integrated, predictable and standards-based networks, Time-Sensitive Networking (TSN) and Wireless TSN (WTSN) have been developed [2,3].

The capabilities of TSN are defined as part of the IEEE 802.1 standards [4] family, the goal of which is to enable converged networks by ensuring time synchronization, determinism, low latency, low jitter, and reliability for time-critical communications, also in conjunction with cellular networks [5,6]. The rise of the Industrial Internet of Things (IIoT) has further increased interest in converged unified networks, which offer the scalability and flexibility required for IIoT applications [7]. Hence, TSN and WTSN are fundamental in overcoming the unresolved challenges of these networks, facilitating more integrated and reliable communication systems [3,8,9].

In particular, WTSN is relevant in autonomous systems and Artificial Intelligent (AI)-driven robotic systems, where the need for deterministic and low latency communication is amplified. In fact, autonomous robots rely on predictable and timely data exchange to maintain stable control loops, ensure safe motion execution, and operate in a reliable manner in dynamic environments. Instead, in AI-driven robotics systems, perception, prediction, and control algorithms are highly sensitive to timing irregularities. In such contexts, even small packet delivery variations or high latency values can propagate through the decision making algorithm, affecting the task execution and the system responsiveness. For example, AI-based models typically require input data to be sampled and delivered at regular intervals. Hence, if these data streams are delayed, arrive irregularly or are partially lost during transmission, the model receives temporally inconsistent information, which can compromise inference quality affecting the behavior of the robot.

Traffic scheduling is a fundamental capability of TSN, encapsulated within the IEEE 802.1Qbv standard [10], commonly referred to as the Time-Aware Shaper (TAS). This standard is designed to optimize network performance by establishing precise transmission schedules that categorize various classes of traffic according to their delay and jitter sensitivity. By allocating dedicated time slots for time-sensitive traffic flows, the 802.1Qbv standard ensures that critical data is transmitted with minimal latency and interference, thus enhancing the reliability and efficiency of network communications. Hence, such a standard enables deterministic and low-latency communication, over standard Ethernet or Wi-Fi, by implementing a time-based scheduling mechanism.

To delve into the traffic scheduling capabilities of WTSN, we focus on an application involving on a UGV that has to follow a trajectory, whose waypoints are periodically transmitted by another device via Wi-Fi. We focus on Wi-Fi among the different wireless technologies specifically to employ the TSN feature, thanks to the significant improvements in the latest versions of the standard [11]. In fact, when dealing with mobile robots, the choice of employing WTSN is necessary to allow the UGV to move freely in the environment without limitations due to wired cabling.

Despite the controller employed in this study being deterministic, the performed analysis can be extended also to adaptive or learning based controllers. In fact, these algorithms depend on input features regularly sampled in order to update the internal model, refine predictions, or adapt policies online. Hence, timing imperfections, caused by misconfigured traffic windows or synchronization errors, can disrupt these observation patterns, leading to degraded inference quality, unstable policy updates, or reduced trajectory accuracy. By examining how scheduling inaccuracies in IEEE 802.1Qbv affect the timely reception of trajectory related data, the study provides insights that hold for both deterministic and learning based robotic controllers.

In practice, in this paper we investigate the impact of traffic scheduling on trajectory tracking accuracy in a wireless network scenario involving a UGV that has to track a desired time-dependent trajectory. The UGV receives trajectory waypoints from a secondary station, highlighting the importance of precise timing in trajectory tracking. Based on a simulation model, this study investigates the impact of the application of imprecise traffic scheduling on trajectory tracking. In detail, different sizes of the scheduled windows in a scenario where the frames are sent with constant periodicity are tested by analyzing the repercussions on trajectory accuracy. Then, by adding an offset on the clock of the secondary station, which affects the synchronization of traffic scheduling between the devices involved, the effects of traffic shaping on such a scenario are evaluated. Consequently, we offer a mathematical model to predetermine the length of RT and BE windows, ensuring accurate RT frame reception despite synchronization offsets between devices. Unlike our previous work in  [12], where we developed a controller to correct synchronization errors, this study directly addresses device offsets to prevent any impact on RT frame reception.

The paper is structured as follows. Section 2 provides an overview of the concept of traffic scheduling, highlighting the relevance of the IEEE 802.1Qbv standard. Section 3 describes the experimental setup, and Section 4 presents and discusses the results of the experimental sessions, also providing the mathematical formulation to establish the length of the RT and BE windows a priori to analyze the number of packets correctly received. Section 6 concludes the paper.

## 2. Background and Motivation

The impact of time synchronization errors on system performance has already been widely studied in various fields, particularly in areas such as sensor data fusion and localization accuracy. In sensor data fusion, a variety of sensors, i.e., GPS, IMUs, LIDAR, cameras, and ultrasonic sensors, collect data at different rates and times. Without proper synchronization, these discrepancies can lead to data mismatches and poor decision-making, as the system may process misaligned or outdated information. In [13], the mathematically modeled time synchronization errors in sensor fusion and target tracking performance are analyzed, highlighting how increasing synchronization errors worsen the tracking accuracy and overall sensor network performance. In [14], it is demonstrated how the accuracy of sensor fusion algorithms is limited by intrinsic sensor noise and by the quality of time synchronization of the sensors that provide the Syncline model to determine which synchronization mechanisms should be used to obtain higher accuracy. Instead, on the effect of time-synchronization errors on positioning, several works have already been presented. In [15], an indoor positioning technique based on UWB Time Difference of Arrival (TDoA) equipment has been optimized to perform time synchronization with timestamps and to demonstrate how this optimization reduces the average positioning error. In [16], instead, the accuracy of the proposed and improved time synchronization and measurement of the error of the TDoA–based location detection technology are evaluated, proving how the TDoA–based location measurement error is improved due to the reduction in the time synchronization error. In addition to these applications, accurate time synchronization is a critical requirement in a variety of other fields, including industrial automation, automotive systems [17], environmental monitoring [18], and power management [19], where the coordination of distributed devices is essential to ensure the timely execution of tasks. Hence, time synchronization is not a new topic, and continues to attract research interest due to the growing demand for precise synchronization in various applications and the introduction of new standards that progressively improve the accuracy of synchronization [20]. In this regard, the IEEE 802.1AS standard, a generalized profile of PTP [21], has revolutionized synchronization by achieving, in some cases, nanosecond precision, a notable improvement over previous methods such as the Network Time Protocol (NTP) [22] and the Simple Network Time Protocol (SNTP) [23], which typically provide millisecond-level precision [24]. The IEEE 802.1AS standard plays a crucial role in the TSN domain, both for wired and wireless networks, emphasizing its importance in modern real-time networked systems. Studies have showcased the applicability of time synchronization in diverse scenarios, as discussed in [2,25]. In [2], which highlights the capabilities of WTSN within a collaborative robotic workcell comprising two robotic arms, simulating a material handling scenario, the focus lies on exploring various configurations and devising measurement methodologies to correlate wireless network performance. Instead, in [25], the analysis performed focuses again on a collaborative task, detailing a methodology to align the Quality of Service (QoS) requirements of the application layer of the Robotic Operating System 2 (ROS2) and the Data Distribution Service (DDS) middleware with the link layer transport using WTSN.

IEEE 802.1Qbv has been extensively researched and implemented due to its ability to handle various types of traffic and minimize latency for RT traffic despite interference. For example, in [26], the end-to-end latency of time-sensitive flows is examined using the TAS mechanism, as defined by the IEEE 802.1Qbv standard. In [27], online incremental routing and scheduling schemes are introduced for the IEEE 802.1Qbv TAS, focusing on multipath routing and non-zero jitter bounds to enhance scheduling flexibility and success. In [28], the study explores how scheduling precedence affects the performance of TAS in IEEE 802.1Qbv networks, particularly in terms of end-to-end delays. It finds that prioritizing traffic with shorter time slots can effectively reduce delays. Instead, in [29], TAS and frame preemption are compared, highlighting that while time-aware shaping provides precise data exchange, it demands complex scheduling, especially in multi-hop networks.

Despite this, there has been a lack of studies that focus on determining the optimal lengths of traffic windows to ensure that RT traffic remains unaffected and is consistently transmitted exactly within the designated RT window.

It is important to emphasize that the effectiveness of traffic scheduling in TSN is heavily dependent on the quality of synchronization among all involved devices. While IEEE 802.1AS is, in theory, capable of guaranteeing sub-10ns synchronization offsets, in practice the accuracy depends on several factors, including the communication medium, the tuning and configuration of the synchronization algorithm, and the computational and hardware capabilities of the devices. For instance, Ref. [8] clearly shows that synchronization accuracy strongly depends on whether the medium is Ethernet or Wi-Fi, as well as on the gains applied to the servo control algorithm regulating synchronization.

Moreover, while TSN features, such as traffic scheduling and synchronization, are already supported in hardware by low-cost microcontrollers for wired networks [30], this is not yet the case for wireless TSN. Current WTSN support is largely limited to mid- to high-end devices and network interfaces, with little or no availability in compact, resource-constrained platforms such as microcontrollers or systems-on-chip. This poses a significant limitation for lightweight and low-power applications, such as drones or mobile robots, where resource efficiency is critical.

Despite this lack of native hardware support, IEEE 802.1AS and IEEE 802.1Qbv can still be implemented in software, for example, through software timestamping mechanisms such as ptp4l [31]. However, software-based approaches introduce a non-negligible overhead that can degrade synchronization accuracy to offsets ranging from hundreds of microsecond up to tens of milliseconds [32]. Consequently, in heterogeneous TSN/WTSN networks comprising devices with widely varying capabilities, synchronization accuracy may be far from optimal. This can lead to impairments in transmission, control, coordination, and ultimately in the motion accuracy of mobile robots.

Unlike the works discussed above, our research focuses on evaluating how synchronization impairments and non-ideal IEEE 802.1Qbv configurations affect trajectory tracking in a simulated mobile robotics environment. In particular, we investigate how the selection of scheduling window lengths, under both ideal and non-ideal synchronization conditions, influences control performance. Based on this analysis, we propose a methodology to identify, in advance, the most suitable RT and BE scheduling window lengths. The goal is to ensure correct frame reception and reliable task completion, even in the presence of desynchronization.

## 3. Experiment Setup

### 3.1. Communication Architecture

The experimental setup is based on a master–slave architecture designed to guide a UGV along a predefined path. In this configuration, the master system, referred to as the Waypoint Generator (WG), is responsible for creating the target trajectory. This trajectory is represented as a sequence of waypoints, where each waypoint specifies not only the desired position and heading angle of the UGV but also the exact, predetermined time at which it must be reached.

The WG generates and transmits these waypoints to the UGV, which operates as the slave. Upon receiving a waypoint, the UGV computes the required linear and angular velocities to reach the specified target. By continuously processing and following the sequence of waypoints, the UGV is able to execute the entire trajectory as dictated by the master.

In practice, the UGV consists of two independent subsystems, both implemented within the ROS framework: the UGV Controller and the UGV itself. The UGV Controller is implemented as an ROS node responsible for receiving waypoints from the WG and computing the appropriate linear and angular velocities, *v* and ω, required to reach them. The details of how *v* and ω are computed are provided in Section 3.4. The UGV itself (i.e., the simulated actuators) is implemented by another ROS entity that uses Gazebo to simulate both the robot’s physical dynamics and the surrounding environment. The velocities *v* and ω computed by the UGV Controller are published via a dedicated ROS topic, which the Gazebo-based UGV entity subscribes to in order to execute the motion.

Since the target applications involve mobile robots, communication between the WG and the UGV Controller would typically rely on a wireless link such as IEEE 802.11 in a real-world deployment. In this study, however, communication is handled in a simulated environment through a virtual network layer, which emulates the characteristics of a wireless channel. This approach provides a controlled testing environment where network conditions and configurations can be flexibly manipulated and analyzed. Specifically, the network layer is implemented using the Linux Traffic Control (tc) subsystem, in combination with network namespaces and virtual Ethernet (veth) pairs. This setup enables the isolation of network contexts and the emulation of diverse communication scenarios. In practice, the WG and the UGV Controller reside on the same Linux-based PC but operate within separate namespaces to simulate two isolated hosts. A veth pair connects the namespaces, with each endpoint configured with multiple transmit and receive queues to emulate realistic behavior. IP addresses within the same subnet are assigned to the veth interfaces, thereby enabling inter-namespace communication. The exact implementation details and configuration of these tools are discussed in [33].

It is important to clarify that while the target application domain is WTSN, the experimental validation employs a virtualized network environment based on veth pairs. This methodological choice was deliberately made to isolate and quantify the impact of traffic scheduling (IEEE 802.1Qbv) and time synchronization errors that are the core logical components of the standard, without the confounding variables of the physical wireless channel (e.g., fading, contention, or random backoffs). By abstracting the physical layer, we ensure that the observed trajectory deviations are strictly attributable to scheduling misalignments and synchronization offsets, rather than being shadowed by generalized channel noise. Nevertheless, it has to be noted that computational and OS-level variability (e.g., interrupt latency, context switching, and kernel-to-user space copying) is natively emulated, as the experiments rely on the actual Linux network stack and kernel-space scheduling. This ensures that the scheduling logic is tested against the realistic jitter of a non-real-time operating system.

**Figure 1:**
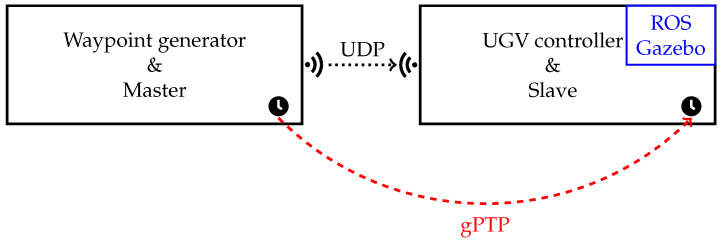
Architecture configuration.

**Figure 2:**
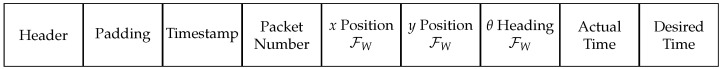
Structure of the UDP frame.

Waypoints are transmitted from the WG to the UGV Controller using the User Datagram Protocol (UDP), as shown in Figure 1. Each UDP frame sent by the WG consists of 256 bytes, with the structure depicted in Figure 2. The frame begins with a header containing the source and destination addresses, UDP frame length, and checksum. This is followed by a padding section of four bytes set to zero to prevent protocol encoding errors. Subsequently, the frame includes a timestamp and a packet number $pkg_{ID}$. Finally, the payload contains the information required by the slave to compute the velocities, namely the desired position (x,y) and heading angle θ, all expressed in the World Frame FW, the actual transmission time $t_{gen}$ at which the frame is sent, and the desired time $t_{des}$ at which the UGV must reach (x,y,θ). The desired time is computed by adding 10ms to $t_{gen}$.

As discussed earlier, after receiving a UDP frame, the UGV Controller calculates the appropriate velocities and publishes them to the relevant ROS topic for the actuator subsystem.

A fundamental aspect of this setup is time synchronization. The WG and UGV must maintain a consistent notion of time to ensure that each waypoint is reached at the intended instant. In real-world scenarios, this is typically achieved using the IEEE 802.1AS standard, which enables precise time synchronization over Ethernet and Wi-Fi networks. The synchronization process relies on the exchange of timing messages between a Grand Master (GM), acting as the reference clock, and the slave devices, which adjust their local clocks accordingly.

In the present case, synchronization is also simulated. Since the WG and UGV processes run on the same virtualized system, they inherently share the same system clock, thereby providing perfect synchronization by default. Nevertheless, Linux networking tools allow the introduction of controlled impairments in order to mimic realistic synchronization errors and limitations. This capability enables a more faithful evaluation of system performance under conditions resembling those encountered in real deployments.

Crucially, these synchronization impairments were introduced as fixed, static offsets rather than stochastic fluctuations typical of a running IEEE 802.1AS protocol. This methodological choice was intended to perform a precise parametric sensitivity analysis. By treating the clock offset as a controlled variable, we can rigorously quantify the direct relationship between the magnitude of synchronization error and the resulting trajectory deviation. This effectively establishes explicit design bounds regarding the synchronization accuracy required for safe operation, which would be difficult to isolate in a fully stochastic simulation.

This setup enabled controlled and reproducible testing of ROS communication performance under varying network conditions, supporting analysis of QoS and synchronization impacts.

All simulations have been run on a PC with an 11th Generation Intel Core i7-1165G7 processor and using Ubuntu 20.04 with ROS Noetic and Gazebo 11.15.1. The simulation environment is initialized using an ROS launch file which loads an empty Gazebo world, with use_sim_time enabled and the Gazebo GUI active. Instead, the UGV is simulated in Gazebo using the TurtleBot3 Burger robot, instantiated from the turtlebot3_burger_for_autorace URDF/Xacro description, that is the standard TurtleBot3 Autorace model. The TurtleBot3 Burger robot, at the beginning of the simulation, is located at (x=0,y=0,z=0) with an initial yaw angle of 1.107rad. The communication and control pipeline is handled by two nodes: a UDP server, running outside ROS, and a UDP receiver, acting also as an ROS node. A UDP sender program is used to transmit trajectory waypoints. Instead, the second node, that is a UDP receiver node receives the waypoints, computes the corresponding linear and angular velocities, and publishes them to /cmd_vel, thereby driving the robot inside Gazebo. Another ROS node is added to subscribe to /odom, published by the Gazebo simulation. This node aims to record the actual position and orientation of the UGV, allowing a direct comparison between the commanded and executed trajectories proposed in Section 4. All these ROS nodes communicate through the ROS master.

### 3.2. Generation of the Trajectory Waypoints

The UGV follows a desired trajectory defined by a sequence of waypoints, ensuring that it reaches, at the predetermined target time $t_{des}$, the specified position and orientation (x,y,θ).

The WG generates the trajectory based on the pattern described in Equation (1),

(1) $$\begin{array}{c} x=\sin(\omega t)\\ y=\sin(2\omega t)\\ \theta =tan^{-1}\left(\frac{dx(t)}{dy(t)}\right) \end{array}$$

where ω=2πf with $f=1s^{-1}$ and t= 10,000$\cdot pkg_{ID}\cdot 10^{-9}$, which is expressed in seconds and depends on the UDP frame number $pkg_{ID}$.

The trajectory defined in Equation (1) was chosen to avoid abrupt step changes in orientation. In fact, abrupt changes in orientation would imply discontinuities in angular velocity and, consequently, unrealistically high (theoretically infinite) angular accelerations when using open-loop motion commands. In practice, this would lead to actuator saturation or kinematically unfeasible commands, introducing large tracking errors unrelated to communication effects. Such errors would overwhelm the results and obscure the contribution of network-induced issues, which is the primary focus of this study. The chosen trajectory represents a well-established benchmark in mobile robotics, as it provides rich and continuous excitation of the system dynamics, thus capturing a representative set of conditions relevant to assessing network-induced effects on motion execution. As shown in the figure, it continuously varies the curvature and motion conditions, requiring the UGV to alternate between quasi-linear motion, moderate curvature transitions, and high-curvature turns at the ends of the loop. This allows us to evaluate the impact of traffic scheduling and packet reception behavior across a wide range of linear and angular velocities within a single experiment, without introducing artificial discontinuities or model violations.

The chosen trajectory is divided into 10,000 waypoints, so the WG generates and sends a UDP frame every 10 ms.

Using a high waypoint density, i.e., 10,000 waypoints, is a deliberate design choice, necessary for precise error isolation. Since the reference trajectory is curved, a low waypoint density would result in a jagged polygonal path due to linear interpolation. Hence, the resulting discretization error would be significant and could override the errors caused by the network under test. So, using a dense waypoint representation ensures a uniform reference, ensuring that any measured deviations can be attributed strictly to network timing and communication issues rather than to trajectory discretization artifacts.

**Figure 3:**
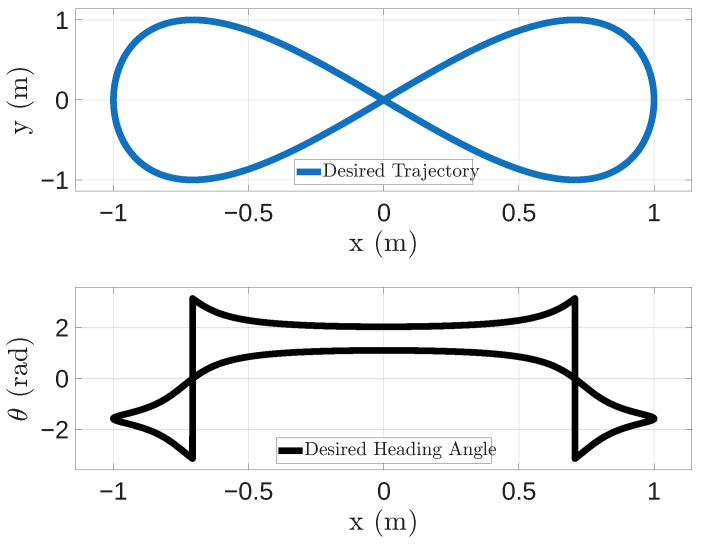
Desired UGV trajectory to track and desired heading angle.

**Figure 4:**
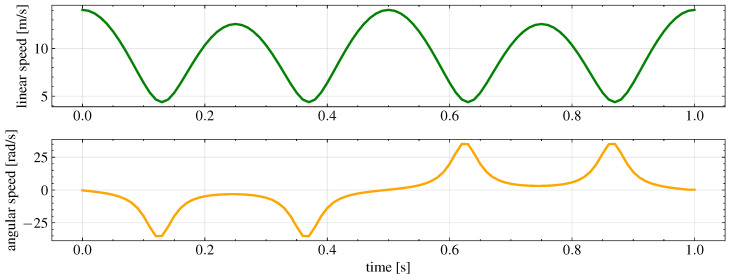
Reference linear and angular velocities associated with the desired trajectory, showing continuous variations in curvature and motion conditions, alternating quasi-linear segments, moderate curvature transitions, and high-curvature turns at the loop extremities.

The final trajectory to be tracked is shown in Figure 3. Instead, the reference linear and angular velocities are shown in Figure 4.

### 3.3. Traffic Scheduling Impairments

We start from the assumption that in general the communication between the WG and the UGV Controller may take place in a non-isolated network, where, in addition to the RT traffic, i.e., the UDP frames containing the waypoints, other types of traffic may be present. In these cases, the coexistence of mixed priority traffic, i.e., RT and BE, needs to be taken into account as it can lead to interference and delays in the transmission of RT frames, which are critical for the accurate execution of the trajectory. To mitigate these issues, traffic scheduling mechanisms such as those defined in the IEEE 802.1Qbv standard can be employed to prioritize RT traffic and ensure its timely delivery.

**Figure 5:**
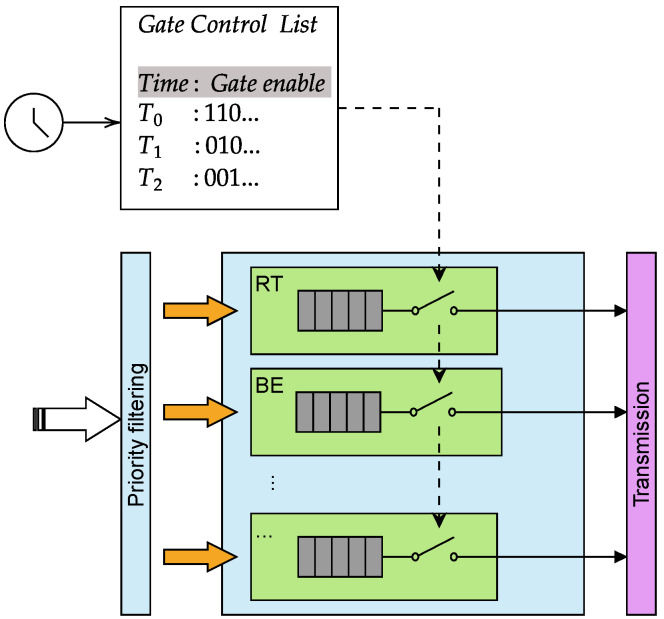
Example of IEEE 802.1Qbv TAS operation.

An intuitive explanation of how IEEE 802.1Qbv TAS works is shown in Figure 5. The mechanism is based on a set of Gate Control Lists (GLs), which define periodic time slots during which specific traffic classes are allowed or prevented from being transmitted. For each cycle, the GCL specifies which queues are enabled for transmission. For instance, during time window $T_{0}$, both RT and BE queues are granted access to the transmission selection process, while other queues remain blocked. In the following window $T_{1}$, only the BE queue is enabled. The figure also highlights the role of priority filtering, which first separates frames into the corresponding per-class queues, and the subsequent gating mechanism, which selectively opens or closes each queue according to the configured schedule. The schedule takes the timing from the synchronized clock shared by all devices in the network via IEEE 802.1AS. It is evident then that if multiple devices are present in the network, they must be synchronized to the same clock to ensure that the gating mechanism operates correctly and consistently across all nodes. Also, the scheduling needs to be accurately configured and shared among the devices to match the traffic patterns and requirements of the application, as misconfigurations can lead to suboptimal performance due to devices overlapping or missing their transmission windows. Moreover, the length of the time windows must be carefully chosen to ensure that all critical RT frames can be transmitted within their designated slots, especially when the traffic generation is periodic.

In the current work, to assess the effects of deterministic scheduling on networked control system performance, IEEE 802.1Qbv (Time-Aware Shaping) is applied to manage the transmission of RT UDP traffic emitted by the WG. In fact, considering the scenario where the waypoints are sent periodically every 10 ms, the primary objective is to investigate how the varying parameters of the GCL, i.e., the timing and duration of transmission windows, affect the ability of the system to meet the stringent temporal constraints required for accurate trajectory tracking.

In detail, Time-aware scheduling was implemented using traffic control tc and the queueing discipline TAPRIO qdisc Linux tools, configured on both veth interfaces, created as explained in Section 3.1. In applying IEEE 802.1Qbv, a fully synchronized configuration is considered, where the clocks of the WG and UGV are synchronized and therefore the TAPRIO schedules shared a common base time using CLOCK_TAI, i.e., the monotonic, leap-second-free timescale used by the gate control scheduler within the Linux kernel to determine precisely when transmission gates open and close, allowing deterministic slot-based transmission.

Within this setup, specific traffic classes were defined using clsact and u32 filters to classify packets based on the port numbers of the source and destination. RT UDP packets generated by the WG were assigned to high-priority time slots, while BE traffic, added through iperf3 Linux tool, and non-deterministic ROS communication, such as topic publication, subscription, and interaction with the ROS master, were assigned lower priority queues.

This experimental setup allowed for a detailed evaluation of how the coexistence of mixed priority traffic, i.e., RT, ROS, and BE, interacts under various IEEE 802.1Qbv GCL configurations, thus providing information on the performance trade-offs and benefits of applying time-aware scheduling to robotic systems operating over shared communication media.

To further evaluate the impact of scheduling on real-time performance, multiple scheduling cycle configurations with varying transmission window alignments were tested. Each scheduling cycle includes a dedicated time window for RT traffic and another for BE and ROS traffic. Varying the time durations of these windows allows us to observe scenarios where the RT packet is generated during its assigned RT window, resulting in immediate transmission with minimal delay (best-case scenario), as well as cases where the packet arrives just after the RT window has closed, forcing it to wait until the RT window of the next cycle opens (worst-case scenario) or to lose the packet. This experimentation highlights how precise alignment between packet generation and transmission windows critically influences the reception of the packets and overall system responsiveness.

In detail, to quantify the delay introduced by the scheduling misalignment, let $T_{cycle}$ denote the duration of the full scheduling cycle, $t_{RT}$ the duration of the RT traffic window in each cycle, and $t_{BE}$ the length of the BE and ROS traffic window in each cycle so that Equation (2) is satisfied.

(2) $$\begin{array}{cc} t_{RT} & <T_{cycle},\\ t_{BE} & <T_{cycle},\\ T_{cycle} & =t_{RT}+t_{BE} \end{array}$$

If a frame, which contains the waypoint information, is generated at time $t_{gen}$, the actual transmission time of the RT frame, that is, $t_{tx}$, depends on its alignment with the RT window.

**Figure 6:**
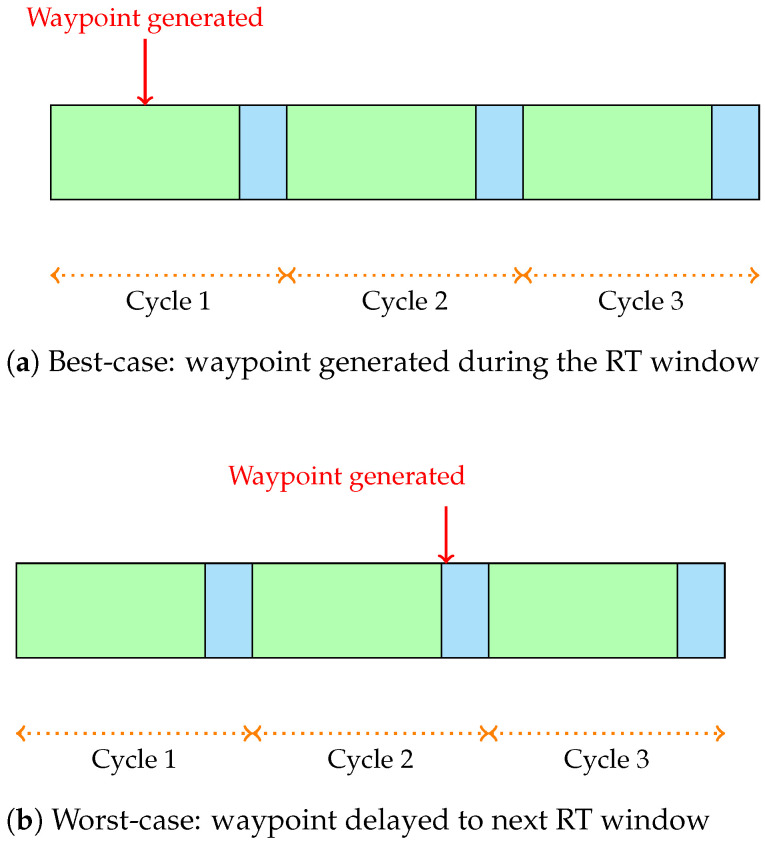
Best- and worst-case alignment of waypoint generation with scheduled RT transmission windows. In green is the RT window, in cyan the BE window, and each cycle is marked with an orange dotted arrow.

In the best-case scenario, as shown in Figure 6a, where the time at which the RT frame is generated is inside the RT window in cycle *k*, i.e., $t_{gen}=t_{RT,start}^{k}+\epsilon$, the scheduling delay is equal to 0 ($\Delta t_{best}=0$) since the frame is transmitted immediately.

Instead, in the worst-case scenario, as shown in Figure 6b, the frame is generated just after the RT window has closed, implying that the frame must wait for the next RT window to be transmitted. This results in a delay of $\Delta t_{worst}\approx T_{cycle}-t_{BE}$. In general, if the frame is generated during the BE window:

(3) $$t_{RT,end}^{k}<t_{gen}<t_{RT,start}^{k+1}$$

hence:

(4) $$t_{gen}\approx t_{RT,end}^{k}+\epsilon$$

with $\epsilon \in [t_{RT,end}^{k},t_{RT,start}^{k+1}]$, then:

(5) $$\Delta t_{worst}\approx T_{cycle}-t_{BE}-\epsilon$$

so that, as ϵ→0:

(6) $$\Delta t_{worst}\approx T_{cycle}-t_{BE}$$

Basically, the worst case depends on how small the RT window is with respect to the cycle duration, since the smaller the RT window is, the longer is the potential wait time for a frame generated just after the RT window closes.

Instead, in addition to considering different scheduling cycle configurations, we have also tested the case where the clocks of the transmitting and receiving devices are not synchronized. In such a scenario, an additional delay can be introduced due to the misalignment between the expected and actual transmission windows, that is, $\Delta t_{offset}$. This misalignment can lead to discrepancies in the timing of packet generation and the availability of transmission opportunities, affecting the overall responsiveness of the system. In detail, if the frame is generated at time $t_{gen}$ according to the clock of the transmitting device, the receiving device receives this at time $t_{gen}^{\prime }=t_{gen}+\Delta t_{offset}$.

In the best case, considering asynchronous clocks, the frame is generated during the RT time window so that $t_{RT,start}^{k}\leq t_{gen}^{\prime }\leq t_{RT,end}^{k}$, which implies that there is no delay in scheduling: $\Delta t_{best}=0$.

Instead, in the worst case, the frame is generated just after the RT window closes $t_{RT,end}^{k}<t_{gen}^{\prime }<t_{RT,start}^{k+1}$, thus obtaining a delay defined as: $\Delta t_{worst}\approx T_{cycle}-t_{BE}-\Delta t_{offset}$.

Hence, clock offset $\Delta t_{offset}$ can change the perceived generation time of a frame, potentially moving it outside the intended RT window and increasing the delay. The impact of unsynchronized clocks is particularly significant when the RT window is small relative to the cycle duration, as even minor offsets can lead to substantial delays.

Taking into account all these considerations and knowing that three different types of traffic are involved in such a setup, i.e., the RT traffic from WG to UGV controller, the BE traffic added to create congestion and the ROS traffic, the Linux tc subsystem has been used to configure the Time-Aware Priority Scheduler (TAPRIO) queuing discipline on both the client interface (UGV Controller, veth1) and server interface (WG, veth4), which reside in separate network namespaces. TAPRIO enables support for IEEE 802.1Qbv (Time-Aware Shaper) thus ensuring deterministic packet transmission by opening and closing gate masks on hardware queues according to a cyclic time schedule.

Packet classification is implemented using the clsact queuing discipline in combination with u32 filters, which identify traffic based on port numbers from the transport layer. Latency-sensitive ROS datagrams (UDP port 12345) and XMLRPC control messages (TCP port 11311) are marked with SKB priority 1, while background traffic, such as iperf3 UDP streams (port 5201) is marked with priority 2. The TAPRIO queueing discipline is configured with two traffic classes, and a map directive assigns priorities 0 and 1 to traffic class 1 (TC1), while all other priorities default to traffic class 0 (TC0). The queues directive then maps TC0 to TX queue 0 and TC1 to TX queue 1, enabling the kernel to schedule packet transmission according to their assigned priority. To emulate realistic network transmission mimicking maximum bandwidth of Wireless Network Interfaces like in [8,25], a Token Bucket Filter (TBF) is applied to shape egress traffic to 1 Gbit/s, with defined burst size and latency parameters. This configuration ensures that time-critical ROS control traffic is transmitted reliably and with bounded latency, even in the presence of high-throughput BE traffic, thereby demonstrating the viability of software-based TSN implementations for real-time robotic and industrial communication over standard Ethernet.

Transmission scheduling is controlled by two schedule entries that alternately open the gates for TC0 and TC1 for specified time intervals. Various configurations of these time windows were tested to assess their impact on RT traffic delivery. Given that each RT frame is generated at fixed intervals of 10 ms, different scenarios were examined. This setup provides TC1, reserved for RT traffic, with a dedicated transmission window, guaranteeing bounded latency and jitter control, while TC0 handles BE and ROS traffic during the remaining portion of the cycle. The goal was to assess latency implications and scheduling behavior under both aligned and misaligned timing conditions. Specifically, the sets of window durations are listed in Table 1.

The base time parameter ensures synchronized schedules across both endpoints using the TAI clock (CLOCK_TAI), essential for coordination in distributed systems.

Furthermore, rather than using a common synchronized base-time, the server’s TAPRIO queuing discipline has been deliberately offset with respect to the client to simulate staggered transmission windows, so that the gate control sequences are temporally misaligned due to the base-time offset. This configuration realistically models distributed systems where perfect time synchronization may not always be achievable and enables the evaluation of how scheduling offsets impact deterministic traffic flows. In this setup, particular emphasis is placed on configuring distinct base-times for the client and server to simulate asynchronous scheduling behavior in a TSN environment. By offsetting base-times, the experiment assesses the resilience of TSN mechanisms under partial synchronization, revealing potential latency implications and jitter behavior in scenarios typical of multi-node robotic or industrial networks. To assess the impact of clock desynchronization between the client and the server on the performance of IEEE 802.1Qbv, two distinct offset values were tested by repeating the same experiments. The offsets considered were 3,000,000 ns and 12,000,000 ns. The smaller offset, being less than the 10ms frame period, allowed evaluation of system behavior under sub-period shifts. The larger offset, corresponding to one full period in scenarios with $t_{BE}=1\;\mathrm{ms}$ and $t_{RT}=11\;\mathrm{ms}$ (or vice versa), was chosen to study the effect of a full-cycle shift. Together, these values enabled assessment of the system under both partial- and full-period offsets.

### 3.4. UGV Controller

The controller has been deployed to improve the analysis of traffic scheduling impacts, particularly focusing on variations in the duration of scheduled windows and cycle time. Specifically, it has been tested to highlight the consequences of inaccurate scheduling in scenarios where RT frames are transmitted deterministically at consistent time intervals.

**Figure 7:**
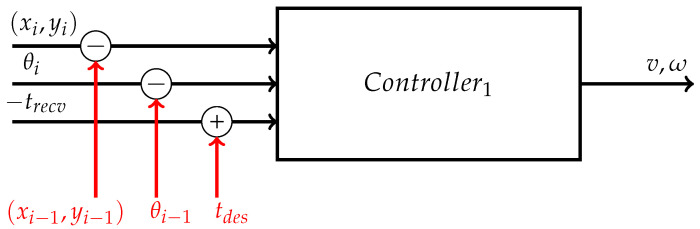
Input and output variables of the controller adopted to correct the offsets between the involved clocks.

The controller used is shown in Figure 7.

It takes as inputs the 2-dimensional coordinates and the heading angle of the next and last waypoints, $\left(x_{i},y_{i},\theta_{i}\right)$ and $\left(x_{i-1},y_{i-1},\theta_{i-1}\right)$, respectively, in addition to the time instant at which the UGV receives the UDP frame, i.e., $t_{recv}$. Hence, it computes the Euclidean distance between the two considered waypoints, i.e., $d_{i}=\sqrt{{\left(x_{i}-x_{i-1}\right)}^{2}+{\left(y_{i}-y_{i-1}\right)}^{2}}$, the difference in heading angle between the two consecutive waypoints, i.e., $\Delta \theta_{i}=\theta_{i}-\theta_{i-1}$, and the time interval available to reach the next waypoint, i.e., $\tau =t_{des}-t_{recv}$.

So, the linear *v* and angular ω velocities are determined as in Equation (7).

(7) $$\begin{array}{cc} v & =\frac{d_{i}}{\tau }\\ \omega & =\frac{\Delta \theta_{i}}{\tau } \end{array}$$

## 4. Results

**Figure 8:**
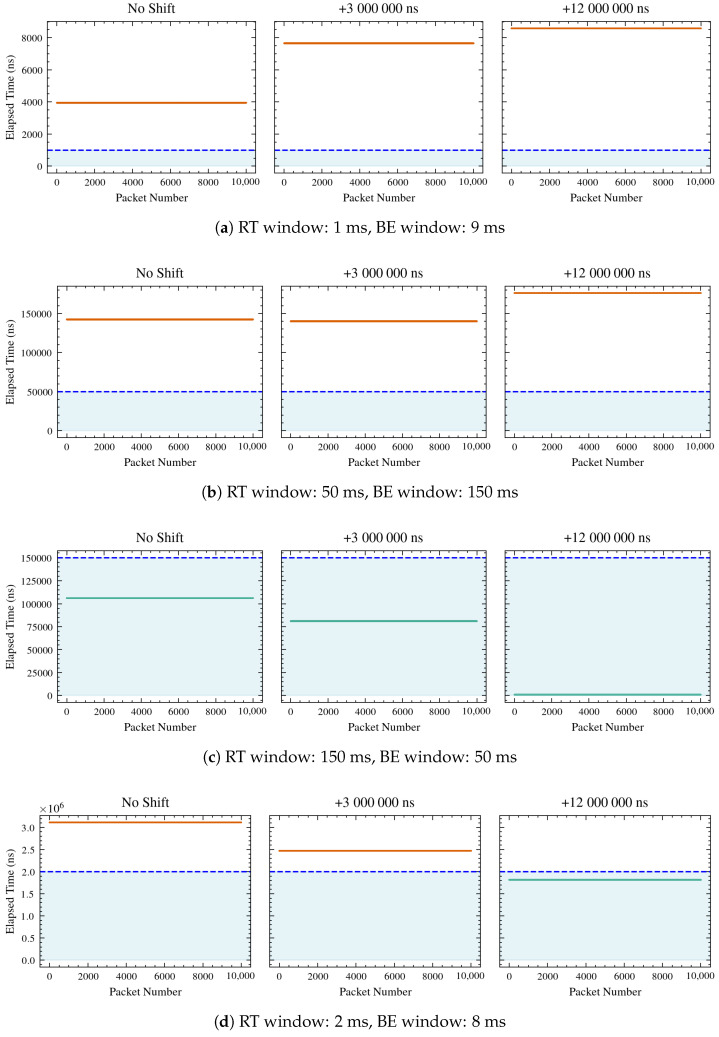

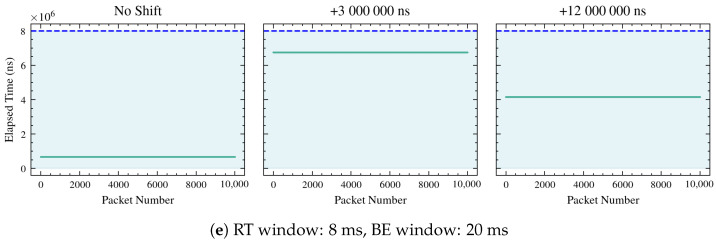
Packet reception timeline for different RT and BE window configurations. Each subfigure corresponds to a specific RT/BE window allocation and shows packet reception for three UGV clock shifts (0, 3, and 12 ms). The green shaded region denotes the RT transmission window. Horizontal lines represent received packets: packets received within the RT window are shown in green, while packets falling outside the RT window are shown in red.

**Figure 9:**
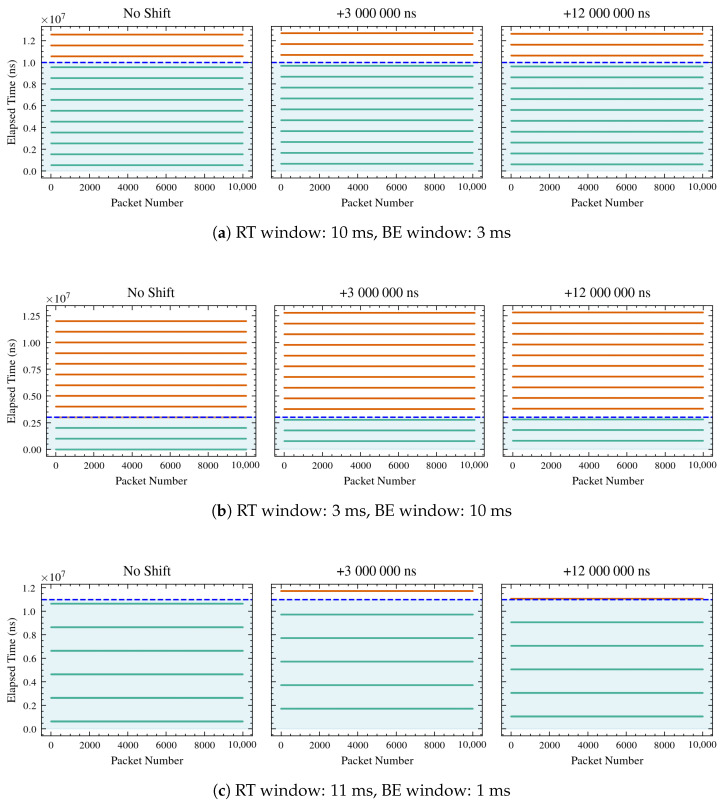

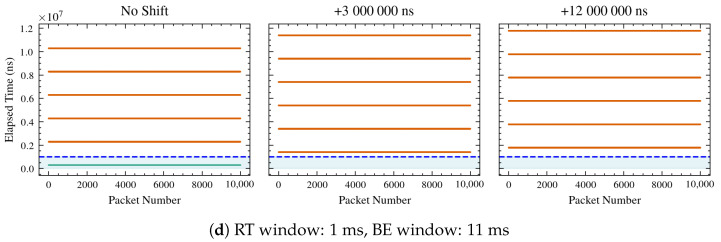
Continuation of Figure 8. Packet reception timelines for additional RT and BE window configurations, further illustrating the impact of window sizing on correct frame reception and packet loss under different UGV clock shifts. The green shaded region denotes the RT transmission window. Horizontal lines represent received packets: packets received within the RT window are shown in green, while packets falling outside the RT window are shown in red.

Considering the setup described in Section 3.1, the WG sends to the UGV one frame every 10 ms, hence since the simulation lasts 10 s, the overall number of packets that should be sent and received is 10,001. However, trying different lengths of RT and BE windows, and hence lengths of cycle time, it has been observed that different numbers of packets are correctly received, as shown in Figure 8 and Figure 9. In detail, only those falling inside the RT window on both the sender and receiver sides are correctly sent and received, depicted in green in the plots, while those depicted in red are dropped.

To evaluate the temporal correctness of frame transmissions in a cyclic time-aware scheduling system, we define a formal method to determine whether individual frames fall within a designated RT transmission window. Let $T_{base}$ denote the base time in nanoseconds at which the first schedule cycle begins, and let $T_{cycle}$ be the cycle period, which defines the interval at which the transmission schedule repeats. The RT window is defined to occupy the first $t_{RT}$ nanoseconds of each cycle, where $t_{RT}\leq T_{cycle}$. Each transmitted frame is indexed by i∈{0,1,…,N−1}, where *N* is the total number of frames. Let $T_{start}$ be the time at which the first frame is transmitted and *P* the fixed period between successive frame transmissions. Hence, the time at which the *i*-th frame is sent is given by:

(8) $$T_{frame}\left(i\right)=T_{start}+i\cdot P$$

In some configurations, a phase shift $T_{shift}$ may be applied to the schedule, for example to align the cycle with other system events. In that case, the effective base time becomes the following:

(9) $$T_{eff}=T_{base}+T_{shift}$$

To determine whether a frame is transmitted during the RT window of a given cycle, we compute the time elapsed into the cycle at the moment of transmission. This is calculated as follows:

(10) $$T_{elapsed}\left(i\right)=\left(T_{frame}\left(i\right)-T_{eff}\right)\;\mathrm{mod}\;T_{cycle}$$

A frame is considered *correctly scheduled* if it is transmitted within the RT window, i.e., if $T_{elapsed}\left(i\right)<t_{RT}$. The total number of correctly scheduled frames across all *N* transmissions is therefore given by:

(11) $$\mathrm{Correct}\;\mathrm{Count}=\sum_{i=0}^{N-1}\left[T_{elapsed}\left(i\right)<t_{RT}\right]$$

where the expression $T_{elapsed}\left(i\right)<t_{RT}$ is 1 if the condition is true and 0 otherwise. This formulation allows us to systematically evaluate the alignment of periodic transmissions with a cyclic time-aware schedule and quantify the effectiveness of scheduling configurations such as those used with TAPRIO.

Hence, to ensure that all *N* frames are transmitted within the designated RT transmission window, we must ensure that for all i∈{0,1,…,N−1}, the elapsed time in the cycle at which the *i*-th frame is transmitted satisfies the following:

(12) $$\left(T_{frame}\left(i\right)-T_{eff}\right)\;\mathrm{mod}\;T_{cycle}<t_{RT}$$

Recalling Equations (8) and (9), the condition becomes:

(13) $$\left(T_{start}+i\cdot P-T_{base}-T_{shift}\right)\;\mathrm{mod}\;T_{cycle}<t_{RT}$$

So, we must choose the RT window size $t_{RT}$ such that:

(14) $$t_{RT}\geq \max_{i=0}^{N-1}\left[\left(T_{start}+i\cdot P-T_{base}-T_{shift}\right)\;\mathrm{mod}\;T_{cycle}\right]$$

subject to the constraint:

(15) $$t_{RT}\leq T_{cycle}$$

This guarantees that all frames are transmitted always within the RT window, avoiding dropping any frames.

Even if the proposed mathematical framework refers to a scenario involving only one UGV, it can be naturally extended to multi-robot scenarios, where timing communication is even more critical. In detail, considering the case in which *J* agents are involved, each agent (j∈1,…,J) is associated with a traffic flow characterized by its own transmission parameters and, eventually, its own effective timing offset. Hence, to guarantee that all frames of all *J* agents are transmitted within their respective RT windows, the following condition, obtained by applying the reasoning previously described for one robot to multiple ones, must hold for every agent (*j*) and for all frames ($i\in 0,1,\ldots ,N_{j}-1$):

(16) $$\left(T_{start}^{\left(j\right)}+i\cdot P^{\left(j\right)}-T_{base}-T_{shift}^{\left(j\right)}\right)\;\mathrm{mod}\;T_{cycle}<t_{RT}^{\left(j\right)}$$

subject to the constraint:

(17) $$t_{RT}^{\left(j\right)}\leq T_{cycle}$$

Additionally, since the multiple agents involved would share the same cycle and link, a new constraint must be imposed to ensure the timing correctness. In fact, in such case, the sum of all RT window allocations and of the BE window does not have to exceed the $T_{cycle}$:

(18) $$\sum_{j=1}^{J}t_{RT}^{\left(j\right)}+t_{BE}\leq T_{cycle}$$

Hence, while in [12], we introduced a controller designed to compensate for time synchronization errors, enabling a UGV to successfully complete its tasks despite potential timing discrepancies, the current study shifts focus to mitigating system errors within a single device using the IEEE 802.1Qbv standard. Considering the deterministic nature of packet transmission, we aimed to introduce a new mathematical technique to formalize a cross layer relationship that is not explicitly captured in the existing literature. In IEEE 802.1Qbv systems, the configuration of the GCL is typically treated as a network layer scheduling problem, whereas the generation of periodic frames and the accumulation of synchronization error belong to the application and system layers, respectively. The formulation presented here provides a unified analytical framework that links these three layers by expressing how application level transmission instants map onto network level gate openings under bounded clock drift. Hence, the equations not only allow us to check whether a periodic event falls within a periodic window, but they also yield a deterministic rule for dimensioning the RT window such that all frames are guaranteed to be transmitted, even in the presence of synchronization error. This enables us to compute the minimum RT window duration required to ensure zero packet loss for a given cycle time, transmission period, and clock offset. This is achieved by knowing the cycle time, the initial time instant when packets begin transmission, and their periodicity. With this information, we can predetermine the appropriate values for the RT and BE windows, ensuring that all packets are received accurately. This method allows for precise configuration of transmission windows, which improves the reliability and efficiency of packet reception on the device.

Hence, the number of expected frames sent and received correctly and the number of frames effectively received for each simulation are given in Table 2.

The observed packet counts match the expected counts, obtained by Equation (11), in all shifts (0 ms, 3 ms, and 12 ms), demonstrating that the performance of the system aligns with the mathematical formulation provided.

In detail, configurations such as 1000/10,000, 50,000/200,000, and 5,000,000/10,000,000 show perfect matches with zero packets expected and zero packets observed in all shifts. Configurations like 150,000/200,000 and 8,000,000/10,000,000 consistently show 10,001 packets expected and observed in all shifts. The configuration 10,000,000/13,000,000 shows a slight discrepancy in the 3 ms shift, with 7694 packets expected but 7693 observed. This minor difference suggests a potential issue in the accuracy of packet reception under certain conditions. The configuration 1,000,000/12,000,000 shows expected and observed counts dropping to zero at shifts of 3 ms and 12 ms, despite having 1667 packets with no clock shifts. This suggests that certain shifts may lead to complete packet loss, which could be due to system limitations or errors in handling time shifts.

In the context of this study, the scenario with an RT window length of 9000 ns is not depicted. This is due to the constraints imposed by $T_{cycle}=$ 10,000 ns, which results in a BE traffic window of only 1000 ns. Such a short duration is insufficient for the ROS nodes to initiate communication with the ROS master. Specifically, within 1000 ns, the ROS nodes cannot establish the necessary connection, causing the simulation to fail to start. This limitation underscores also the importance of adequate BE window length to ensure successful communication and simulation initiation in systems utilizing ROS. The failure in this specific configuration is driven by the guard band mechanism of the Time-Aware Shaper (TAPRIO), which calculates frame transmission feasibility based on this constant rate. The experiment empirically demonstrates that the intrinsic processing overhead of the Linux kernel and ROS middleware exceeds 1000 ns; consequently, the scheduler determines that the handshake packets cannot be transmitted within the remaining open gate duration and drops them. This result provides a practical lower bound for window sizing in software-based WTSN implementations.

**Figure 10:**
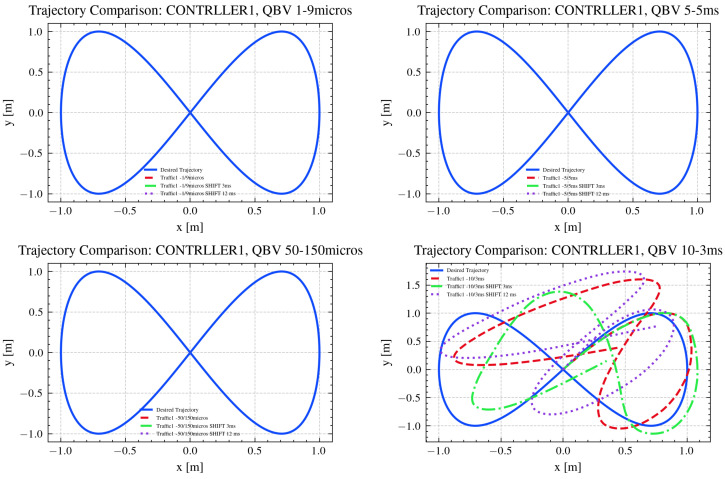

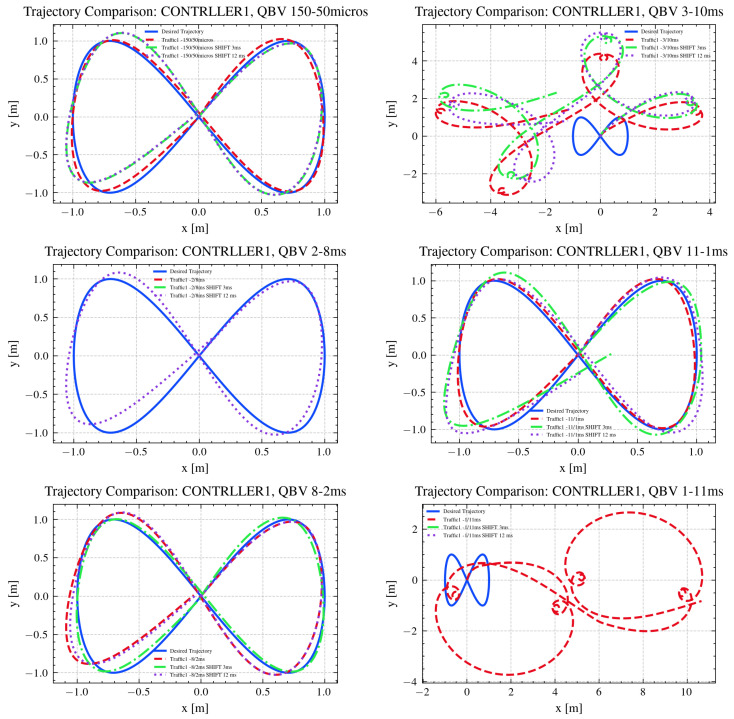
Trajectories tracked under the different tested scenarios.

The tracked trajectories are depicted in Figure 10, while the mean absolute errors for each tracked trajectory, with respect to the desired one, are shown in Table 3.

As shown in Table 2, frame reception is strongly influenced by both the RT window length and the temporal shift applied between the two clocks, thus causing different errors in trajectory tracking, as observed in Table 3. Three operating regimes can be distinguished. When the RT window is too short or misaligned with the frame generation cycle, no frames are received, preventing trajectory tracking. In such cases, the error values remain at nominal levels (approximately 0.637 m in position and 1.571 rad in orientation), reflecting system inactivity. When the RT window is properly synchronized with the processing and communication dynamics of the system, all frames are successfully received. Under these conditions, the system operates with optimal timing, minimizing latency and jitter. Consequently, the mean absolute trajectory errors in *x*, *y*, and θ remain low and consistent across different temporal shifts, indicating accurate and stable tracking. Between these two extremes, partial frame reception occurs when the RT window only partially overlaps with the valid transmission period or when temporal shifts introduce misalignment. In this regime, missing or delayed frames degrade trajectory reconstruction, leading to larger errors and reduced tracking precision. The extent of degradation depends on the fraction of lost frames and the degree of clock misalignment. Notably, when the RT window is set to 3,000,000 ns (with or without clock shifts), 2308 frames are correctly received, and when it is 1,000,000 ns (without any shift), 1667 frames are correctly received. In both cases, a clear increase in trajectory errors, in particular in *x* and *y*, can be observed. These results confirm that as the number of successfully received packets decreases, the position and orientation errors increase accordingly, consistent with the data presented in Table 3. Overall, the results in Table 2 and Table 3 demonstrate a clear relationship between frame reception quality and tracking accuracy. Reliable performance is achieved only when the RT window configuration enables full frame reception, whereas partial or total loss of frames leads to significant increases in trajectory error.

## 5. Propagation of Time Synchronization Uncertainties on the Positioning

As discussed in the previous sections, time synchronization is a key factor for several reasons. Indeed, as shown in the previous section, possible disalignment in the clocks of the two devices can lead to a misalignment in the scheduling of the RT and BE windows, which can cause delays in the transmission of RT frames. This can result in increased latency and jitter, which can negatively impact the performance of real-time applications in terms of following the desired trajectory. In addition to that, when the synchronization is correctly established, the uncertainty in the time synchronization, due to crystal tolerances, temperature oscillation, timestamping resolution, and other factors, can propagate to the positioning of the UGV. This is because the UGV relies on accurate timing information to determine its position and orientation in space. If there is uncertainty in the time synchronization, this can lead to errors in the position and orientation estimates, which can affect the UGV’s ability to navigate and perform tasks accurately.

It is important to note that the experimental analysis in the previous sections focused on the macroscopic effects of traffic scheduling, specifically, preventing packet loss and large transmission delays through correct window sizing. Once these scheduling constraints are met, the system effectively operates in a quasi-delay-free state regarding packet delivery. However, real-world IEEE 802.1AS implementations still introduce microscopic, stochastic timing uncertainties (e.g., clock jitter and crystal drift) that are computationally expensive to simulate directly in the control loop. Therefore, this section complements the simulation by mathematically modeling these residual synchronization uncertainties. So in this section, we address this issue by quantifying the impact of temporal synchronization uncertainties on UGV positioning. Specifically, we focus on an idealized scenario to better isolate the effect of uncertainty in the time synchronization on UGV positioning. Specifically, we assume perfect trajectory knowledge and no delays or jitter due to schedule misalignment. Under these controlled conditions, the only source of uncertainty is the temporal error introduced by the synchronization process. This simplification allows us to quantify how synchronization errors propagate through the UGV kinematic model.

We consider the UGV to be a unicycle model, whose state is defined by the position in the 2D plane, i.e., *x* and *y*, and the heading angle θ. The UGV is controlled by setting the linear velocity *v* and the angular velocity ω. The state of the UGV evolves according to the following kinematics equations:

(19) $$\begin{array}{cc} x_{t} & =x_{t-1}+R\left(\sin\left(\theta_{t-1}+\omega \Delta t\right)-\sin\left(\theta_{t-1}\right)\right)\\ y_{t} & =y_{t-1}-R\left(\cos\left(\theta_{t-1}+\omega \Delta t\right)-\cos\left(\theta_{t-1}\right)\right)\\ \theta_{t} & =\theta_{t-1}+\omega \Delta t \end{array}$$

where $R=\frac{v}{\omega }$ is the turning radius and Δt is the integration time step for the model. Recalling Equation (7):

(20) $$\begin{array}{cc} v & =\frac{\sqrt{{\left(\bar{x_{t}}-x_{t-1}\right)}^{2}+{\left(\bar{y_{t}}-y_{t-1}\right)}^{2}}}{\tau }\\ \omega & =\frac{\bar{\theta_{t}}-\theta_{t-1}}{\tau }\\ R & =\frac{v}{\omega }=\frac{\sqrt{{\left(\bar{x_{t}}-x_{t-1}\right)}^{2}+{\left(\bar{y_{t}}-y_{t-1}\right)}^{2}}}{\bar{\theta_{t}}-\theta_{t-1}} \end{array}$$

where $\left(\bar{x_{t}},\bar{y_{t}},\bar{\theta_{t}}\right)$ is the desired pose at time *t* and τ is the time interval available to reach the next waypoint, i.e., $\tau =t_{des}-t_{recv}$, with $t_{des}$ being the desired time instant at which the UGV should reach the next waypoint and $t_{recv}$ the time instant at which the UGV receives the UDP frame containing the next waypoint.

Here, notice that we are distinguishing between the desired pose, i.e., $\left(\bar{x_{t}},\bar{y_{t}},\bar{\theta_{t}}\right)$, and the actual pose, i.e., $\left(x_{t},y_{t},\theta_{t}\right)$, of the UGV. The desired pose is the one that the UGV should reach at time *t* according to the trajectory planned by the WG, while the actual pose is the one that the UGV effectively reaches at time *t* according to its kinematics model. Since we are not considering any closed-loop control on the UGV position, the actual pose can differ from the desired one as a result of uncertainties in the input variables of the model. In particular, as stated before, we are interested in the uncertainty on the time synchronization, so in the model, the only source of uncertainty is $t_{recv}$. Indeed, $t_{recv}$ is a sample of the internal clock of the UGV, which is synchronized with the WG clock through the PTP protocol. However, the synchronization process brings on the slave clock an uncertainty $u(t_{s})$ [34]. On the slave side clock, we suppose that there is any other source of uncertainty, like the one associated with the sampling resolution of the clock; therefore, according to the propagation of uncertainty, the uncertainty on $t_{recv}$ is equal to $u(t_{s})$.

As the $t_{des}$ is generated by the WG, which is also considered the master clock for the PTP synchronization, we consider it the ground truth, i.e., it is not affected by any uncertainty.

So the only source of uncertainty for the UGV is the uncertainty on the time synchronization, through the uncertainty on $t_{recv}$.

Combining (19) and (20), we can rewrite the kinematics equations as:

(21) $$F_{t}=\left[\begin{array}{c} x_{t}\\ y_{t}\\ \theta_{t} \end{array}\right]=\left[\begin{array}{c} x_{t-1}+\frac{\sqrt{{\left({\hat{x}}_{t}-x_{t-1}\right)}^{2}+{\left({\hat{y}}_{t}-y_{t-1}\right)}^{2}}}{{\hat{\theta }}_{t}-\theta_{t-1}}\left(\sin\;\left(\theta_{t-1}+\frac{{\hat{\theta }}_{t}-\theta_{t-1}}{\tau }\Delta t\right)-\sin\left(\theta_{t-1}\right)\right)\\ y_{t-1}-\frac{\sqrt{{\left({\hat{x}}_{t}-x_{t-1}\right)}^{2}+{\left({\hat{y}}_{t}-y_{t-1}\right)}^{2}}}{{\hat{\theta }}_{t}-\theta_{t-1}}\left(\cos\;\left(\theta_{t-1}+\frac{{\hat{\theta }}_{t}-\theta_{t-1}}{\tau }\Delta t\right)-\cos\left(\theta_{t-1}\right)\right)\\ \theta_{t-1}+\frac{{\hat{\theta }}_{t}-\theta_{t-1}}{\tau }\Delta t \end{array}\right]$$

where F is the function that describes the kinematics of the UGV at time *t*. In other words, F represents the state of the system at time *t*. In particular it depends on the internal state at time t−1, i.e., $\left(x_{t-1},y_{t-1},\theta_{t-1}\right)$, and on the input variables, i.e., the desired pose at time *t*, i.e., $\left({\hat{x}}_{t},{\hat{y}}_{t},{\hat{\theta }}_{t}\right)$, other than on τ.

The uncertainty on the state of the system at time *t* depends on the uncertainties associated with the state as well as on the input variables.

According to the Guide to the Expression of Uncertainty in Measurement (GUM), the propagation of uncertainty is generally addressed through a linear approximation of the measurement model. If a quantity of interest *y* is expressed as a function of a set of input quantities $x_{1},x_{2},\ldots ,x_{n}$, i.e.,

(22) $$y=f(x_{1},x_{2},\ldots ,x_{n}),$$

Then the combined standard uncertainty of *y* can be computed as

(23) $$u^{2}\left(y\right)=\sum_{i=1}^{n}{\left(\frac{\partial f}{\partial x_{i}}\right)}^{2}u^{2}\left(x_{i}\right)+2\sum_{i=1}^{n-1}\sum_{j=i+1}^{n}\frac{\partial f}{\partial x_{i}}\frac{\partial f}{\partial x_{j}}\;u\left(x_{i},x_{j}\right),$$

where $u^{2}\left(x_{i}\right)$ are the variances of the input quantities and $u(x_{i},x_{j})$ are their covariances. This formulation highlights that both the individual uncertainties and the correlations among the inputs contribute to the overall output uncertainty.

In the more general multivariate case, as in this case, it can be generalized in the form

(24) $$P_{t|t-1}=AP_{t-1}A^{⊤}+BQB^{⊤}$$

where $P_{t|t-1}$ is the state covariance at time *t* given the state at time t−1, $P_{t-1}$ is the state covariance at time t−1, and *Q* is the process noise covariance, i.e., the uncertainties associated with the input that are considered time-invariant.

To explain *A* and *B*, let us first define the two vectors

(25) $$\begin{array}{cc} w & ={\left[x_{t-1},y_{t-1},\theta_{t-1}\right]}^{⊤} \end{array}$$

(26) $$\begin{array}{cc} z & ={\left[{\hat{x}}_{t},{\hat{y}}_{t},{\hat{\theta }}_{t},\tau \right]}^{⊤} \end{array}$$

*A* and *B* are defined as

(27) $$\begin{array}{cc} A & =\frac{\partial F}{\partial w} \end{array}$$

(28) $$\begin{array}{cc} B & =\frac{\partial F}{\partial z} \end{array}$$

Basically, *A* is the Jacobian matrix with respect to the state variables w and it describes the propagation of the state covariance from the previous step. The element in row *i* and column *j* contains the partial derivative of the *i*-th state function F with respect to the *j*-th variable of the previous state.

Hence:

(29) $$\begin{array}{c} A=\left[\begin{array}{ccc} A_{11} & A_{12} & A_{13}\\ A_{21} & A_{22} & A_{23}\\ A_{31} & A_{32} & A_{33} \end{array}\right] \end{array}$$

where, defining:

(30) $$\begin{array}{cc} \Delta x & ={\hat{x}}_{t}-x_{t-1}\\ \Delta y & ={\hat{y}}_{t}-y_{t-1}\\ R & =\sqrt{\Delta x^{2}+\Delta y^{2}}\\ \Delta \theta & ={\hat{\theta }}_{t}-\theta_{t-1} \end{array}$$

it holds that:

$$\begin{array}{cc} A_{11} & =1+\frac{-\Delta x\left(-\sin\theta_{t-1}+\sin\left(\frac{\Delta t}{\tau }\Delta \theta +\theta_{t-1}\right)\right)}{\Delta \theta R}\\ A_{12} & =\frac{-\Delta y\left(-\sin\theta_{t-1}+\sin\left(\frac{\Delta t}{\tau }\Delta \theta +\theta_{t-1}\right)\right)}{\Delta \theta R}\\ A_{13} & =\frac{\left(\left(-\frac{\Delta t}{\tau }+1\right)\cos\left(\frac{\Delta t}{\tau }\Delta \theta +\theta_{t-1}\right)-\cos\theta_{t-1}\right)R}{\Delta \theta }+\frac{R\left(-\sin\theta_{t-1}+\sin\left(\frac{\Delta t}{\tau }\Delta \theta +\theta_{t-1}\right)\right)}{{\left(\Delta \theta \right)}^{2}}\\ A_{21} & =-\frac{-\Delta x\left(-\cos\theta_{t-1}+\cos\left(\frac{\Delta t}{\tau }\Delta \theta +\theta_{t-1}\right)\right)}{\Delta \theta R}\\ A_{22} & =1-\frac{-\Delta y\left(-\cos\theta_{t-1}+\cos\left(\frac{\Delta t}{\tau }\Delta \theta +\theta_{t-1}\right)\right)}{\Delta \theta R}\\ A_{23} & =-\frac{\left(\left(-\frac{\Delta t}{\tau }+1\right)\sin\left(\frac{\Delta t}{\tau }\Delta \theta +\theta_{t-1}\right)-\sin\theta_{t-1}\right)R}{\Delta \theta }-\frac{R\left(-\cos\theta_{t-1}+\cos\left(\frac{\Delta t}{\tau }\Delta \theta +\theta_{t-1}\right)\right)}{{\left(\Delta \theta \right)}^{2}}\\ A_{31} & =0\\ A_{32} & =0\\ A_{33} & =1-\frac{\Delta t}{\tau } \end{array}$$

*B*, instead, is the Jacobian matrix with respect to the input variables z quantifying how the uncertainty originating from the new measurements is propagated into the current state. It is defined as follows:

(31) $$\begin{array}{c} B=\left[\begin{array}{ccc} B_{11} & B_{12} & B_{13}\\ B_{21} & B_{22} & B_{23}\\ B_{31} & B_{32} & B_{33} \end{array}\right] \end{array}$$

with:

$$\begin{array}{cc} B_{11} & =\frac{\Delta x\left(-\sin\theta_{t-1}+\sin\left(\frac{\Delta t}{\tau }\Delta \theta +\theta_{t-1}\right)\right)}{\Delta \theta \;R}\\ B_{12} & =\frac{\Delta y\left(-\sin\theta_{t-1}+\sin\left(\frac{\Delta t}{\tau }\Delta \theta +\theta_{t-1}\right)\right)}{\Delta \theta \;R}\\ B_{13} & =\frac{\Delta t\;R\cos(\frac{\Delta t}{\tau }\Delta \theta +\theta_{t-1})}{\tau \;\Delta \theta }-\frac{R\left(-\sin\theta_{t-1}+\sin\left(\frac{\Delta t}{\tau }\Delta \theta +\theta_{t-1}\right)\right)}{{\left(\Delta \theta \right)}^{2}}-\frac{\Delta t\;R\cos(\frac{\Delta t}{\tau }\Delta \theta +\theta_{t-1})}{\tau^{2}}\\ B_{21} & =-\frac{\Delta x\left(-\cos\theta_{t-1}+\cos\left(\frac{\Delta t}{\tau }\Delta \theta +\theta_{t-1}\right)\right)}{\Delta \theta \;R}\\ B_{22} & =-\frac{\Delta y\left(-\cos\theta_{t-1}+\cos\left(\frac{\Delta t}{\tau }\Delta \theta +\theta_{t-1}\right)\right)}{\Delta \theta \;R}\\ B_{23} & =\frac{\Delta t\;R\sin(\frac{\Delta t}{\tau }\Delta \theta +\theta_{t-1})}{\tau \;\Delta \theta }+\frac{R\left(-\cos\theta_{t-1}+\cos\left(\frac{\Delta t}{\tau }\Delta \theta +\theta_{t-1}\right)\right)}{{\left(\Delta \theta \right)}^{2}}-\frac{\Delta t\;R\sin(\frac{\Delta t}{\tau }\Delta \theta +\theta_{t-1})}{\tau^{2}}\\ B_{31} & =0\\ B_{32} & =0\\ B_{33} & =\frac{\Delta t}{\tau }-\frac{\Delta t\;\Delta \theta }{\tau^{2}} \end{array}$$

As can be seen, the matrices *A* and *B* are quite complex due to the non-linear nature of the kinematics equations. Moreover, clearly, both are time variant, since they depend on the state and input variables at each time step. This poses some challenges in assessing the uncertainty propagation. Indeed, it is usually desirable to study asymptotically and in a global fashion the propagation of the uncertainty by analyzing the eigenvalues of the system matrix in such a way as to understand if, given a certain model of the system and a certain model of the input uncertainty, the system is stable or not in terms of uncertainty propagation. However, due to the time-variant nature of *A* and *B*, a global assessment is not possible, but it is possible to analyze the stability along a specific trajectory by computing the uncertainty propagation only locally at each time step.

In this direction, we consider the same trajectory as in the previous section and we compute the uncertainty propagation along it. We consider a time step of Δt= 10 ms. The initial state of the UGV is set to $\left(x_{0},y_{0},\theta_{0}\right)=\left(0,0,0\right)$ and the initial state covariance $P_{0}$ is set to zero, i.e., we assume that the initial position and orientation of the UGV are known with certainty. The process noise covariance *Q* is set to zero for all variables except for τ, which is set to $u^{2}\left(t_{s}\right)$, i.e., the variance of the time synchronization uncertainty on the slave clock.

It is important to emphasize that the modeling assumptions adopted here, specifically the exclusion of sensing noise, actuation errors, and physical constraints, are intentional simplifications. These factors were deliberately omitted to decouple network-induced errors from mechanical or environmental variability. Including them would introduce trajectory deviations that could mask the specific impact of synchronization uncertainty, making it difficult to isolate the contribution of the WTSN timing impairments. However, the proposed Jacobian-based framework is inherently general; additional uncertainty sources (e.g., sensor noise) can be readily incorporated by populating the corresponding diagonal elements of the process noise covariance matrix. We have explicitly chosen to set these terms to zero in this study to maintain a strict focus on the propagation of time synchronization errors.

For the numerical values of $u(t_{s})$, we considered different values from [8,30] which reports the time synchronization uncertainty achieved in different scenarios with different hardware and different communication systems. Numerical values are shown in Table 4.

**Figure 11:**
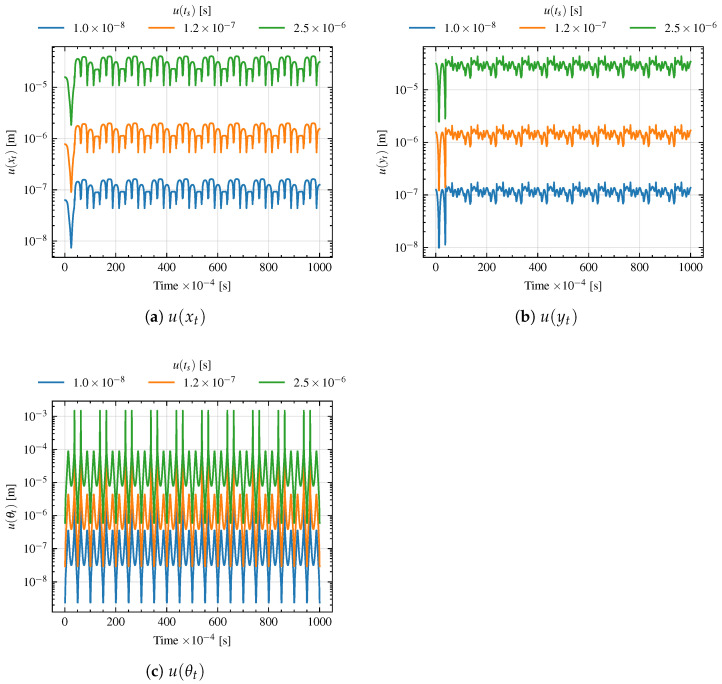
Uncertainties on the UGV position and orientation along the trajectory for different values of time synchronization uncertainty $u(t_{s})$.

The results of the analysis are shown in Figure 11, where the uncertainties on *x*, *y*, and θ along the trajectory are shown for the different values of $u(t_{s})$. As can be seen, and as expected from Equations (29) and (31), the uncertainty on the position and orientation of the UGV is non-constant along the trajectory, but at some point, it remains asymptotically bounded with a certain value. The mean values of the uncertainties along the trajectory are reported in Table 5.

**Figure 12:**
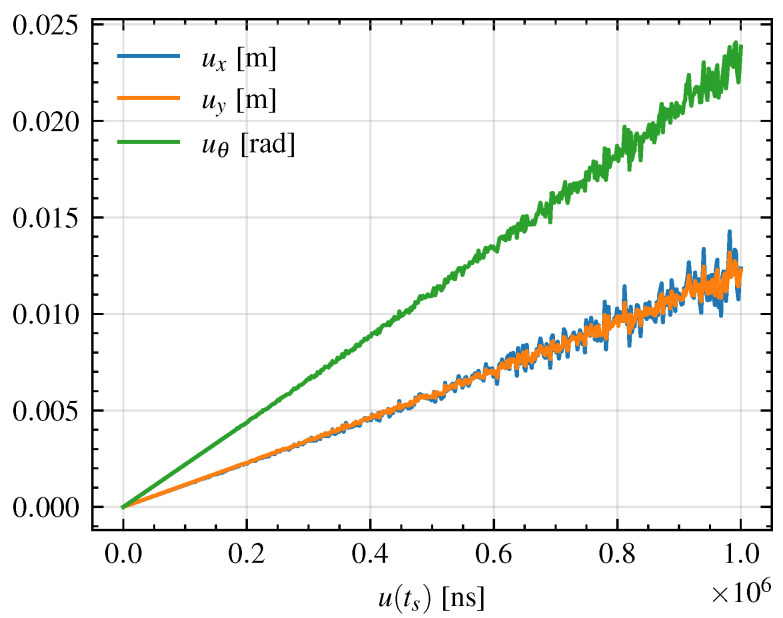
Mean uncertainties on the UGV position and orientation along the trajectory for different values of time synchronization uncertainty $u(t_{s})$.

To generalize the results, in Figure 12, the mean uncertainties on *x*, *y* and θ along the trajectory are shown for different values of $u(t_{s})$, ranging from 1 ns to 1 ms. As can be seen, the uncertainty on the position and orientation of the UGV increases linearly with the uncertainty on the time synchronization. However, especially for values of $u(t_{s})$ greater than 400 μs, some oscillation in the trend is visible. This suggests that, even if globally the uncertainty linearly increases with $u(t_{s})$, locally, for a certain trajectory, the induced uncertainty on u(x),u(y),u(θ) can be smaller with higher values of $u(t_{s})$. Therefore, this behavior suggests that the propagation of the time synchronization uncertainty is trajectory-dependent and not strictly monotonic with respect to $u(t_{s})$. In other words, while on average a higher synchronization uncertainty leads to a higher uncertainty in the vehicle’s state estimation, the local dynamics and geometry of the trajectory, such as curvature, velocity profile, and heading changes, can induce compensatory or amplifying effects on the uncertainty evolution.

This effect can be explained by considering that the uncertainty propagation is governed by the Jacobian matrices *A* and *B*, which depend on both the instantaneous state and control inputs. When the UGV undergoes sections of the trajectory characterized by small curvature or quasi-straight motion, the sensitivity of the state to timing errors is reduced, leading to local plateaus or even decreases in the propagated uncertainty. Conversely, during turns or more dynamic maneuvers, the coupling between linear and angular motion amplifies the uncertainty growth. This interplay makes the overall uncertainty propagation a non-trivial function of the system dynamics, even when the input uncertainty grows monotonically.

From a practical standpoint, this finding highlights the importance of trajectory design and timing synchronization management in systems relying on distributed sensing and control, especially when precise temporal coordination among nodes is required. For instance, in cooperative or swarm robotics applications, where multiple agents share time-stamped measurements or commands, trajectories with frequent directional changes may exacerbate the effects of clock misalignment, leading to degraded estimation accuracy or instability in feedback control.

## 6. Conclusions

This work investigated the role of traffic scheduling in a WTSN-enabled network. Specifically, we analyzed the impact of traffic scheduling in a WTSN-enabled network supporting a UGV tasked with tracking a time-dependent trajectory, where waypoints are transmitted periodically. Our study demonstrated that inadequate scheduling not only reduces the number of successfully received UDP frames but also degrades trajectory tracking accuracy. Our analysis confirmed that proper scheduling is essential for reliable task execution, particularly in mobile robotics scenarios where timing guarantees are critical.

A distinctive contribution of this study is the evaluation of synchronization disturbances and non-ideal IEEE 802.1Qbv configurations, which revealed how desynchronization amplifies control performance degradation. Our results demonstrate that practical disturbances must be carefully considered when implementing TSN over wireless links. Based on these insights, we proposed a methodology for selecting appropriate RT and BE scheduling window lengths. This methodology allows designers to anticipate the impact of timing inaccuracy and configure programming parameters that preserve control performance, even under imperfect conditions.

These results highlight the interaction between synchronization accuracy, traffic scheduling and control stability in the WTSN. In particular, even in scenarios where time synchronization is not possible, traffic scheduling can be implemented in such a way as to ensure the correct execution of the task.

Finally, we acknowledge several simplifications inherent in the experimental design adopted in this study. To isolate and quantify the specific impact of traffic scheduling and synchronization errors on control performance, we employed a controlled simulation environment that idealizes certain physical-layer characteristics, such as wireless channel fading and medium contention, as well as robotic uncertainties, including sensor noise and actuation latency. Moreover, the current evaluation is limited to simulation-based experiments involving a single UGV following a predefined trajectory. While this approach enables a clear separation between network-induced deviations and drifts attributable to mechanical modeling inaccuracies, real-world deployments are likely to exhibit a more complex superposition of these effects. Future work will therefore focus on extending the analysis to hardware-in-the-loop configurations and real-world WTSN implementations, incorporating stochastic environmental and mechanical disturbances, and exploring multi-agent scenarios with multiple mobile robots. In addition, further investigations will consider advanced TSN mechanisms, such as traffic shaping and frame preemption, to provide a more comprehensive assessment of the robustness and applicability of WTSN for safety-critical and resource-efficient control tasks in mobile robotics and beyond.

## Figures and Tables

**Table 1:** List of experiments with specified lengths of the RT and BE windows.

Experiment	RT Length [ns]	BE Length [ns]
1	9000	1000
2	1000	9000
3	150,000	50,000
4	50,000	150,000
5	5,000,000	5,000,000
6	8,000,000	2,000,000
7	2,000,000	8,000,000
8	10,000,000	3,000,000
9	3,000,000	10,000,000
10	11,000,000	1,000,000
11	1,000,000	11,000,000

**Table 2:** Expected and observed success counts for different RT window and cycle configurations under varying UGV clock shifts.

RT Window/Cycle [ns]	UGV Clock Shift					
	0 ms		3 ms		12 ms	
	Expected	Observed	Expected	Observed	Expected	Observed
1000/10,000	0	0	0	0	0	0
50,000/200,000	0	0	0	0	0	0
150,000/200,000	10,001	10,001	10,001	10,001	10,001	10,001
2,000,000/10,000,000	0	0	0	0	10,001	10,001
8,000,000/10,000,000	10,001	10,001	10,001	10,001	10,001	10,001
5,000,000/10,000,000	0	0	0	0	0	0
10,000,000/13,000,000	7693	7693	7694	7693	7693	7693
3,000,000/13,000,000	2308	2308	2308	2308	2308	2308
11,000,000/12,000,000	10,001	10,001	8334	8334	8334	8334
1,000,000/12,000,000	1667	1667	0	0	0	0

**Table 3:** Mean absolute trajectory errors along *x*, *y*, and heading θ for different RT window and cycle configurations under varying UGV clock shifts.

RT Window/Cycle [ns]	Mean Absolute Error for Each Clock Shift								
	x Position [m]			y Position [m]			Heading θ [rad]		
	0 ms	3 ms	12 ms	0 ms	3 ms	12 ms	0 ms	3 ms	12 ms
1000/10,000	0.637	0.637	0.637	0.637	0.637	0.637	1.571	1.571	1.571
50,000/200,000	0.637	0.637	0.637	0.637	0.637	0.637	1.571	1.571	1.571
150,000/200,000	0.808	0.809	0.809	0.806	0.809	0.810	1.556	1.567	1.570
2,000,000/10,000,000	0.637	0.637	0.813	0.637	0.637	0.809	1.571	1.571	1.569
8,000,000/10,000,000	0.817	0.807	0.811	0.806	0.806	0.809	1.561	1.555	1.567
5,000,000/10,000,000	0.637	0.637	0.637	0.637	0.637	0.637	1.571	1.571	1.571
10,000,000/13,000,000	0.785	0.782	0.739	0.901	0.854	0.927	1.585	1.576	1.584
3,000,000/13,000,000	2.762	2.609	2.373	1.845	2.229	2.205	1.482	1.506	1.536
11,000,000/12,000,000	0.809	0.837	0.845	0.808	0.821	0.825	1.563	1.559	1.558
1,000,000/12,000,000	4.850	0.637	0.637	4.725	0.637	0.637	1.644	1.571	1.571

**Table 4:** Time synchronization uncertainties considered for the analysis.

Source	$u(t_{s})$ (ns)
[30]	123
[8]	10
[8]	2500

**Table 5:** Mean uncertainties for different clock uncertainties.

$u(t_{s})$ (ns)	u(x) (m)	u(y) (m)	u(θ) (rad)
10.0 × $10^{-9}$	113.3 × $10^{-9}$	114.4 × $10^{-9}$	218.7 × $10^{-9}$
123.0 × $10^{-9}$	1.4 × $10^{-6}$	1.4 × $10^{-6}$	2.7 × $10^{-6}$
2.5 × $10^{-6}$	28.3 × $10^{-6}$	28.6 × $10^{-6}$	54.7 × $10^{-6}$

## Data Availability

The original contributions presented in this study are included in the article. Further inquiries can be directed to the corresponding author.
